# On the Impact of Entropy Estimation on Transcriptional Regulatory Network Inference Based on Mutual Information

**DOI:** 10.1155/2009/308959

**Published:** 2008-11-26

**Authors:** Catharina Olsen, Patrick E Meyer, Gianluca Bontempi

**Affiliations:** 1 Machine Learning Group, Computer Science Department, Faculty of Science, UniversitÃ© Libre de Bruxelles, CP 212, 1050 Brussels, Belgium

## Abstract

The reverse engineering of transcription regulatory networks from expression data is gaining large interest in the bioinformatics community. An important family of inference techniques is represented by algorithms based on information theoretic measures which rely on the computation of pairwise mutual information. This paper aims to study the impact of the entropy estimator on the quality of the inferred networks. This is done by means of a comprehensive study which takes into consideration three state-of-the-art mutual information algorithms: ARACNE, CLR, and MRNET. Two different setups are considered in this work. The first one considers a set of 12 synthetically generated datasets to compare 8 different entropy estimators and three network inference algorithms. The two methods emerging as the most accurate ones from the first set of experiments are the MRNET method combined with the newly applied Spearman correlation and the CLR method combined with the Pearson correlation. The validation of these two techniques is then carried out on a set of 10 public domain microarray datasets measuring the transcriptional regulatory activity in the yeast organism.

## 1. Introduction

The inference of regulatory networks by modeling dependencies at the transcription level aims at providing biologists with an additional insight about cell activities. This task belongs to the domain of *systems biology* which studies the interactions between the components of biological systems and how these interactions give rise to the function and the behavior of the whole system. This approach differs from the so-called "reductionist approach" that limits its focus to the building blocks of the system without providing a global picture of the cell behavior, as stated in [1]:

"the reductionist approach has successfully identified most of the components and many of the interactions but, unfortunately, offers no convincing concepts or methods to understand how system properties emerg  the pluralism of causes and effects in biological networks is better addressed by observing, through quantitative measures, multiple components simultaneously and by rigorous data integration with mathematical models."

The reverse engineering of transcriptional regulatory networks (TRNs) from expression data is known to be a very challenging task because of the large amount of noise intrinsic to the microarray technology, the high dimensionality, and the combinatorial nature of the problem. Also, a gene-to-gene network inferred on the basis of transcriptional measurements returns only a rough approximation of a complete biochemical regulatory network since many physical connections between macromolecules might be hidden by shortcuts. Notwithstanding, in recent years, computational techniques have been applied with success to this domain, as witnessed by successful validations of the interaction networks predicted by the algorithms [2].

Network inference consists in representing the stochastic dependencies between the variables of a dataset by means of a graph. Mutual information networks are an important category of network inference methods.

Information-theoretic approaches typically rely on the estimation of mutual information (MI) from expression data in order to measure the statistical dependence between genes [3]. In these methods, a link between two nodes is established if it exhibits a significant score estimated by mutual information. The role of the mutual information estimator is therefore essential to guarantee a high accuracy rate. Notwithstanding, few experimental studies about the impact of the estimator on the quality of the inferred network exist [4]. To the best of our knowledge, this paper presents the first comprehensive experimental comparison of several mutual information estimation techniques and state-of-the-art inference methods like MRNET [3], ARACNE [5], and CLR [6]. An additional contribution of this paper is the study of the impact of the correlation estimator (notably Spearman and Pearson) on the mutual information computation once a hypothesis of normality is done. Interestingly enough, the Spearman- and the Pearson-based information estimators emerge as the most competitive techniques once combined with the MRNET and the CLR inference strategies, respectively.

The first part of the experimental session aims at studying the sensitivity to noise and missing values of different discretization, estimation, and network inference methods. For this purpose, a synthetic benchmark is created by means of the SynTReN data generator [7].

In the second part, the techniques which appeared to be the most effective in the synthetic session are assessed by means of a biological microarray benchmark which integrates several public domain yeast microarray datasets.

The outline of the paper is as follows. Section2 reviews the most important mutual information estimators. Section 3 introduces some state-of-the-art network inference algorithms. Section 4 contains the description of the synthetic data generator, the description of the real data setting, and the related discussions of the results. Section 5 concludes the paper.

## 2. Estimators of Information

An information theoretic network inference technique aims at identifying connections between two genes (variables) by estimating the amount of information between them. Different information measures exist in the literature [8]. In this article, we focus on the mutual information measure and the related estimation techniques. Note that, if the estimation technique has been conceived for discrete random variables, a discretization procedure has to be executed before applying the estimation procedure to expression data.

### 2.1. Mutual Information

Mutual information is a well-known measure which quantifies the stochastic dependency between two random variables without making any assumption (e.g., linearity) about the nature of the relation [9].

Let  be a discrete random vector whose th component takes values in the discrete set  of size . The th element of the mutual information matrix (MIM) associated to  is defined by(1)

where the entropy of a discrete random variable  is defined as(2)

and  is the mutual information between the random variables  and .

### 2.2. Entropy Estimation

In practical setups, the underlying distribution  of the variables is unknown. Consequently, the entropy terms in (1) cannot be computed directly but require an estimation. Many different approaches to entropy estimation have been introduced. In this paper, we restrict the choice to the following five estimators: empirical, Miller-Madow, shrink, Pearson and Spearman correlation.

#### 2.2.1. Empirical

Let  be a continuous random variable taking values in the real interval . Suppose the interval is partitioned into  bins, where  denotes the bin index vector,  denotes the number of data points in the th bin, and  stands for the total number of observations.

The empirical estimator, also known as the *maximum likelihood estimator*, is the entropy of the empirical distribution(3)

It has been shown in [10] that the asymptotic bias of this estimator amounts to(4)

#### 2.2.2. Miller-Madow

The Miller-Madow estimator [10] corrects the biased empirical estimator by removing the estimated bias term from it (4):(5)

This estimator reduces the bias of (3) without increasing its variance.

#### 2.2.3. Shrink

The shrink estimator [8] combines two different estimators, one with low variance and one with low bias, by using the weighting factor :(6)

Let(7)

be the value minimizing the mean square function [8]. It has been shown in [11] that the optimal  is given by(8)

#### 2.2.4. Pearson Correlation

Correlation is a statistic measuring the strength and the direction of the linear relationship between two random variables. The Pearson correlation between two random variables  and  is defined as(9)

Correlation takes values in , where  denotes a linear relation between the variables  and . If the variables are independent, the correlation is equal to zero while the opposite is not necessarily true (e.g., nonlinear dependency).

It can be shown that correlation and mutual information are related if the joint distribution is normal.

Let(10)

be the density of a multivariate Gaussian variable  with mean  and covariance matrix . The entropy of this distribution is given by(11)

where  is the determinant of the covariance matrix [12]. The mutual information between two variables  and  is then given by(12)(13)

where  is the Pearson's correlation.

Since the functional relation (13) between the mutual information and the correlation is a monotone function, it is sufficient to use  when computing this value.

The Pearson correlation can be estimated from the measurements  and  of two genes  and  by the following equation:(14)

#### 2.2.5. Spearman Correlation

The Spearman rank correlation coefficient is a special case of the Pearson correlation in which the data are converted to rankings before calculating the coefficient.

The Spearman correlation can be calculated using (14), where the terms  and  are replaced by their respective ranks. Note that the Spearman rank correlation coefficient generalizes the Pearson correlation coefficient by being able to detect not only linear relationships between the variables but also any kind of monotone relation without making any assumptions about the distributions of the variables. 

### 2.3. Discretization Methods

The mutual information estimators in Sections 2.2.1, 2.2.2, and 2.2.3 apply to discrete random variables. In order to use them for continuous random variables, a discretization step is required. The two most widely used methods for discretization are the equal width and the equal frequency methods [13].

*Equal Width.* This discretization method partitions the domain of  into  subintervals of equal size. As a consequence, the number of data points in each bin is likely to be different.

*Equal Frequency.* This method divides the interval  into  subintervals, each containing the same number of data points. It follows that subinterval sizes are typically different.

The number of subintervals should be chosen so that all bins contain a significant number of samples. In [14], the authors propose to use , where  is the total number of samples.

## 3. Network Inference Algorithms

The network inference proceeds in two steps. In the first step, the mutual information matrix is calculated. In the second step, the chosen algorithm is applied to the mutual information matrix in order to compute a score that is used to weigh the links between network nodes.

### 3.1. The Mrnet Method

The MRNET method [3] is based on the maximum relevance/minimum redundancy (MRMR) feature selection technique [15]. This iterative selection technique chooses at each step, among the least redundant variables, the one having the highest mutual information with the target.

The method ranks the set of inputs according to a score which is the difference between the mutual information with the output variable  (maximum relevance) and the average mutual information with the previously ranked variables (minimum redundancy). The network is inferred by deleting all edges whose score lies below a given threshold.

Direct interactions should be well ranked whereas indirect interactions should be badly ranked. In the first step, variable  which has the highest mutual information to the target  is selected. The second selected variable  will be the one with a high information  to the target and at the same time a low information  to the previously selected variable.

In the next steps, given a set  of selected variables, the criterion updates  by choosing the variable that maximizes the score(15)

which can be described as a relevance term minus a redundancy term.

For each pair , the algorithm returns two scores  and  and computes the maximum of the two. All edges with a score below a given threshold are then deleted.

### 3.2. The Aracne Method

The algorithm for the reconstruction of accurate cellular networks (ARACNEs) [5] is based on the data processing inequality [16]. This inequality states that if the interaction between  and  depends on , then(16)

The algorithm assigns a weight to each pair of nodes which is equal to the mutual information between the variables. Then, the minimal mutual information between three variables is computed, and eventually the edge with the lowest value is interpreted as an indirect connection and removed if the difference between the two lowest weights is above a given threshold.

### 3.3. The Clr Method

In the context likelihood or relatedness (CLR) algorithm [6], the mutual information is calculated for each pair of variables. Then, a score related to the empirical distribution of these MI values is computed. In particular, instead of considering the information  between two variables  and , the algorithm takes into account the score , where(17)

and  and  are, respectively, the mean and the standard deviations of the empirical distribution of the mutual information values .

## 4. Experiments

This section contains two parts. In the first part, several inference methods and estimators are applied to synthetic datasets with different noise and missing values configurations. The aim of this part is to identify the best combination of estimator and inference method. Once the assessment on the synthetic benchmark is done, the best performing techniques are then applied to a biological problem. The aim of this second experiment is to assess the capability of the algorithms of discovering interactions of the yeast transcriptome uniquely on the basis of expression data.

All computations were carried out using the R-package MINET [17] (http://cran.r-project.org/web/packages/minet). This recently introduced package allows the use of three different inference methods, namely, ARACNE [5], CLR [6], and MRNET [3].

The following entropy estimators are also made available to calculate the mutual information: empirical, Miller-Madow, shrink, and Pearson correlation.

Note that, in order to apply the first three estimators to expression data, two different discretization methods are implemented: equal frequency and equal width discretization with default size .

### 4.1. Synthetic Data

#### 4.1.1. Network Generation

The synthetic benchmark relies on several artificial microarray datasets generated by the SynTReN generator [7]. This simulator emulates the gene expression process by adopting topologies derived from subnetworks of E.coli and S.cerevisiae networks. Interaction kinetics are modeled by nonlinear differential equations based on Michaelis-Menten and Hill kinetics.

We used the SynTReN generator to create twelve benchmark datasets whose number  of samples and number  of genes are detailed in Table 1.

**Table 1:** Generated datasets Number of genes , number of samples .

No.	Dataset	Source net		
1	ecoli_300_300	E.coli	300	300
2	ecoli_300_200	E.coli	300	200
3	ecoli_300_100	E.coli	300	100
4	ecoli_300_50	E.coli	300	50

5	ecoli_200_300	E.coli	200	300
6	ecoli_200_200	E.coli	200	200
7	ecoli_200_100	E.coli	200	100
8	ecoli_200_50	E.coli	200	50

9	ecoli_100_300	E.coli	100	300
10	ecoli_100_200	E.coli	100	200
11	ecoli_100_100	E.coli	100	100
12	ecoli_100_50	E.coli	100	50

#### 4.1.2. Introducing Missing Values

In order to study the impact of missing values, expression values were removed from the generated datasets. The number of missing values is distributed according to the  distribution with parameters  and . The maximal allowed number of missing values is a third of the entire dataset. This distribution was utilized, instead of the uniform distribution, because the latter one could have favored the empirical estimator.

#### 4.1.3. Setup

For each experiment, ten repetitions were carried out. Each dataset was analyzed using three inference methods (i.e., MRNET, ARACNE, and CLR) and the following estimators: Pearson correlation, empirical, Miller-Madow, shrink, and the Spearman correlation coefficient. The empirical, the Miller-Madow, and the shrink estimator were computed applying the equal width and the equal frequency discretization approaches. Furthermore, the computation was carried out with and without additive Gaussian noise (having 50% variance of the observed values). Each of these setups was also assessed with introduced missing values.

#### 4.1.4. Validation

Network inference algorithms infer either the presence or the absence of an edge for each pair of nodes. Similarly to classification, we define the possible outcomes of inference as follows. A true positive (TP) occurs when an edge is correctly predicted as existing, a false positive (FP) occurs when a nonexisting edge is inferred, true negative (TN) occurs when a nonexisting edge is not inferred, and false negative (FN) occurs when an existing edge is not detected.

Once the numbers of TP, FP, TN, and FN are computed, we can measure *precision* and *recall*(18)

Precision measures the fraction of real edges among the ones classified as positive while recall quantifies the fraction of real edges that are correctly inferred.

A weighted harmonic average of precision and recall is returned by the -score [18]:(19)

which attains its maximum value  when the returned network is without any error.

To validate the simulation's results, the maximal -score was computed for each experiment. Using a paired *t*-test, the maximal -scores were then compared and statistically validated.

#### 4.1.5. Discussion of Results

The results of the synthetic benchmark are collected in Table 2 which returns the -score for each combination of inference method, mutual information estimator, and nature of the dataset (noisy versus not noisy, complete versus missing data). Note that the maximal -score is highlighted together with the -scores which are not significantly different from the best.

**Table 2:** MINET results: *noise* stands for Gaussian additive noise, *NA* for missing values, *eqf* for equal frequency, and *eqw* for equal width In bold face maximum -scores and significantly not different values.

Method		MRnet			
Estimator		No noise, no NA	Noise, no NA	No noise, NA	Noise, NA
Pearson		0.2006	0.1691	**0.1790**	**0.1611**
Spearman		0.3230	**0.1771**	0.1464	0.1333

Emp	eqf	**0.3420**	0.1551	0.1136	0.0868
Emp	eqw	0.2028	0.1650	0.1036	0.0822
MM	eqf	**0.3396**	0.1524	0.1140	0.0924
MM	eqw	0.1909	0.1592	0.1068	0.0883
Shr	eqf	0.3306	0.1506	0.1150	0.0788
Shr	eqw	0.1935	0.1574	0.1090	0.0839

		Aracne			

Pearson		0.1117	0.1082	0.1054	**0.1069**
Spearman		**0.1767**	**0.1156**	**0.1167**	**0.1074**

Emp	eqf	**0.1781**	0.1042	0.0993	0.0765
Emp	eqw	0.1287	0.1082	0.0892	0.0727
MM	eqf	**0.1786**	0.1032	0.0985	0.0783
MM	eqw	0.1217	0.1049	0.0931	0.0767
Shr	eqf	0.1736	0.1000	0.1009	0.0697
Shr	eqw	0.1152	0.1045	0.0898	0.0717

		CLR			

Pearson		**0.2242**	**0.1941**	**0.2231**	**0.1911**
Spearman		**0.2197**	**0.1915**	0.1806	0.1582

Emp	eqf	0.2123	0.1729	0.1847	0.1397
Emp	eqw	0.2098	0.1724	0.1799	0.1327
MM	eqf	0.2128	0.1729	0.1860	0.1427
MM	eqw	0.2083	0.1723	0.1845	0.1384
Shr	eqf	0.2096	0.1670	0.1864	0.1311
Shr	eqw	0.2030	0.1659	0.1822	0.1333

We analyze the results according to four different aspects: the impact of the estimator, the impact of the discretization, the impact of the inference algorithm, and the influence of sample and network size.

The section concludes with the identification of the best combination of inference algorithm and estimator.

*Impact of The Estimator.* In case of complete datasets with no noise, the empirical and the Miller-Madow estimators with equal-frequency binning lead to the highest -scores for the MRNET and the ARACNE inference methods. The Spearman correlation is not significantly different from the best, in case of ARACNE, and is close to the best in case of MRNET. The CLR method is less sensitive to the estimator, and the best result is obtained with the Pearson correlation.

In case of noisy data or missing value (NA) configurations, the Pearson correlation and the Spearman correlation lead to the highest -score for all inference methods. A slight better accuracy of the Pearson correlation can be observed in presence of missing values. The Spearman correlation outperforms the other estimators in MRNET and ARACNE when complete yet noisy datasets are considered. In CLR, Pearson and Spearman correlations lead the ranking without being significantly different.

*Impact of The Discretization.* In case of complete datasets with no noise, the equal frequency binning approach outperforms the equal width binning approach for all discrete estimators. The gap between the two discretization methods is clearly evident in MRNET and less striking in ARACNE and CLR. In case of noisy or missing data configurations, differences are attenuated.

*Impact of The Inference Algorithm.* In case of complete datasets with no noise, the MRNET inference technique outperforms the other algorithms.

The situation changes in presence of noisy or missing values. Here, CLR appears to be the most robust by returning the highest -scores for all combinations of noise and missing values.

**Figure 1:**
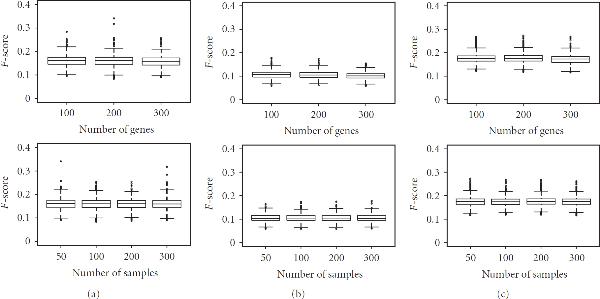
**(First row) Mean -scores and standard deviation with respect to number of genes (Second row) Mean -scores and standard deviation with respect to number of samples**. For all, 10 repetitions with additive Gaussian noise of 50% with full datasets (no missing values). Inference methods: (a) MRNET, (b) ARACNE, and (c) CLR.

*Impact of Number of Sample and Network Sizes.* The role of network size is illustrated in Figure 1 (first row) which shows how the -score decreases as long as the network size increases. This behavior can be explained by the increasing difficulty of recovering a larger underlying network in front of an increasing dimensionality of the modeling task.

In Figure 1 (second row), the values of the -score seem not to be influenced substantially by the number of samples.

*Conclusion*. A concise summary of the previously discussed results is displayed in Table 3 which averages the accuracy over the different data configurations.

It emerges that the most promising combinations are represented by the MRNET algorithm with the Spearman estimator and the CLR algorithm with the Pearson correlation. The former seems to be less biased because of its good performance in front of nonnoisy datasets while the latter seems to be more robust since it is less variant in front of additive noise.

**Table 3:** For each method and estimator, the mean over the four different setups: no NA, no noise; no NA, noise; NA, no noise; NA noise. In bold face the best mean -score.

Estimator		Method		
		MRnet	Aracne	CLR
Pearson		0.1775	0.1081	**0.2081**
Spearman		**0.1950**	**0.1285**	0.1863

Emp	eqf	0.1744	0.1145	0.1774
Emp	eqw	0.1384	0.0997	0.1737
MM	eqf	0.1746	0.1147	0.1786
MM	eqw	0.1363	0.0881	0.1759
Shr	eqf	0.1688	0.1111	0.1735
Shr	eqw	0.1360	0.0953	0.1711

### 4.2. Biological Data

The second part of the experimental session aims to assess the performance of the two selected techniques once applied to a real biological task.

We proceeded by (i) setting up a dataset which combines several public domain microarray datasets about the yeast transcriptome activity, (ii) carrying out the inference with the two selected techniques, and (iii) assessing the quality of the inferred network with respect to two independent sources of information: the list of interactions measured by means of an alternative genomic technology and a list of biologically known gene interactions derived from the TRANSFAC database.

#### 4.2.1. The Dataset

The dataset was built by first normalizing and then joining ten public domain yeast microarray datasets, whose number of samples and origin is detailed in Table 4. The resulting dataset contains the expression of 6352 yeast genes in 711 experimental conditions.

**Table 4:** Number of samples and bibliographic references of the yeast microarray data used for network inference.

Dataset	Number of samples	Origin
1	7	[19]
2	7	[20]
3	77	[21]
4	4	[22]
5	173	[23]
6	52	[24]
7	63	[25]
8	300	[25]
9	8	[26]
10	20	[27]

#### 4.2.2. Assessment by Chip-Chip Technology

The first validation of the network inference outcome is obtained by comparing the inferred interactions with the outcome of a set of ChIP-chip experiments. The ChIP-chip technology, detailed in [28], measures the interactions between proteins and DNA by identifying the binding sites of DNA-binding proteins. The procedure can be summarized as follows. First, the protein of interest is cross-linked with the DNA site it binds to, then double-stranded parts of DNA fragments are extracted. The ones which were cross-linked to the protein of interest are filtered out from this set and reverse cross-linked. Also, their DNA is purified. In the last step, the fragments are analyzed using a DNA microarray in order to identify gene-gene connections. For our purposes, it is interesting to remark that the ChIp-chip technology returns for each pair of genes a probability of interaction. In particular we use, for the validation of our inference procedures, the ChIp-chip measures of the yeast transcriptome provided in [29].

#### 4.2.3. Assessment by Biological Knowledge

The second validation of the network inference outcome relies on existing biological knowledge and in particular on the list of putative interactions in Saccharomyces cerevisiae published in [30].

This list contains 1222 interactions involving 725 genes, and in the following we will refer to this as the Simonis list.

#### 4.2.4. Results

In order to make a comparison with the Simonis list of known interactions, we limited our inference procedure to the  genes contained in the list.

The quantitative assessment of the final results is displayed by means of receiver operating characteristics (ROCs) and the associated area (AUC). This curve compares the true positive rate (TPR) to the false positive rate (FPR) which are defined as follows:(20)

Note that this assessment considers as true only the interactions contained in the Simonis list.

**Figure 2:**
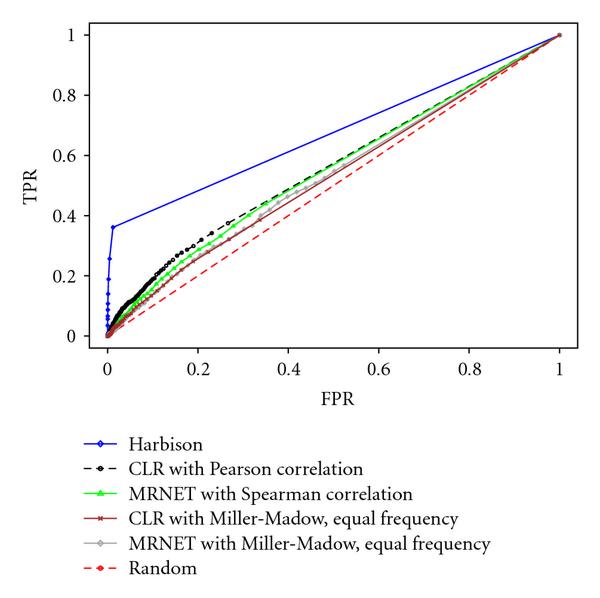
**ROC curves: Harbison network, CLR combined with Pearson correlation, MRNET with Spearman correlation, CLR combined with the Miller-Madow estimator using the equal frequency discretization method, MRNET with Miller-Madow using equal frequency discretization and random decision**.

Figure 2 displays the ROC curves, and Table 5 reports the associated AUC for the following techniques: the ChIP-chip technique, the MRNET-Spearman correlation combination, the CLR-Gaussian combination, the CLR-Miller-Madow combination, the MRNET-Miller-Madow combination, and the random guess.

**Table 5:** AUC: Harbinson, CLR with Gaussian, MRNET with Spearman, CLR with Miller-Madow, MRNET with Miller-Madow.

	AUC
Harbison	0.6632
CLR Pearson	0.5534
MRNET Spearman	0.5433
MRNET Miller-Madow	0.5254
CLR Miller-Madow	0.5207

A first consideration to be made about these results is that network inference methods are able to be significantly better than a random guess also in real biological settings. Also the two combinations which appeared to be the best in synthetic datasets confirmed their supremacy over the Miller-Madow-based techniques also in real data.

However, the weak, though significative, performance of the networks inferred from microarray data requires some specific considerations.

(1) With respect to the ChIP-chip technology, it is worth mentioning that the information coming from microarray datasets is known to be less informative than the one coming from the ChIP-chip technology. Microarray datasets remain nowadays however more easily accessible to the experimental community, and techniques able to extract complex information from them are still essential for system biology purposes.

(2) Both the microarray dataset we set up for our experiment and the list of known interactions we used for assessment are strongly heterogeneous and concern different functionalities in yeast. We are confident that more specific analysis on specific functionalities could increase the final accuracy.

(3) Like in any biological validation of bioinformatics methods, the final assessment is done with respect to a list of putative interactions. It is probable that some of our false positives could be potentially true interactions or at least deserve additional investigation.

## 5. Conclusion

The paper presented an experimental study of the influence of the information measure and the estimator on the quality of the inferred interaction network. The study concerned both synthetic and real datasets.

The study on synthetically generated datasets allowed to identify two effective techniques with complementary properties. The MRNET method combined with the Spearman correlation appeared to be effective mainly in front of complete and accurate measures. The CLR method combined with the Pearson correlation was ranked as the best one in the case of noisy and missing values.

The experiments on real microarray data confirmed the potential of these inference methods and showed that, though in presence of noisy and heterogeneous datasets, the techniques are able to return significative results.
